# Effects of a paddling-based high-intensity interval training prescribed using anaerobic speed reserve on sprint kayak performance

**DOI:** 10.3389/fphys.2022.1077172

**Published:** 2023-01-04

**Authors:** Geng Du, Tao Tao

**Affiliations:** ^1^ Sports Training Department, Wuhan Sports University, Wuhan, China; ^2^ College of Sport, Huzhou University, Huzhou, China

**Keywords:** oxygen uptake, power output, physiological response, athletic performance, intermittent exercise

## Abstract

The aim of this study was to investigate physiological and performance adaptations to high-intensity interval training (HIIT) prescribed as a proportion of anaerobic speed reserve (ASR) compared to HIIT prescribed using maximal aerobic speed (MAS). Twenty-four highly trained sprint kayak athletes were randomly allocated to one of three 4-weak conditions (N = 8) (ASR-HIIT) two sets of 6 × 60 s intervals at ∆%20ASR (MAS-HIIT) six 2 min paddling intervals at 100% maximal aerobic speed (MAS); or controls (CON) who performed six sessions/week of 1-h traditional endurance paddling at 70%–80% maximum HR. A graded exercise test was performed on a kayak ergometer to determine peak oxygen uptake (V̇O_2peak_), MAS, V̇O_2_/HR, and ventilatory threshold. Also, participants completed four consecutive upper-body wingate tests to asses peak and average power output. Significant increases in V̇O_2peak_ (ASR-HIIT = 6.9%, MAS-HIIT = 4.8%), MAS (ASR-HIIT = 7.2%, MAS-HIIT = 4.8%), ASR (ASR-HIIT = −25.1%, MAS-HIIT = −15.9%), upper-body Wingate peak power output and average power output (*p* < 0.05 for both HIIT groups) were seen compared with pre-training. Also, ASR-HIIT resulted in a significant decrease in 500-m $\left(-1.9\%\right)$ , and $1,000-m\;\left(-1.5\%\right)$ paddling time. Lower coefficient of variation values were observed for the percent changes of the aforementioned factors in response to ASR-HIIT compared to MAS-HIIT. Overall, a short period of ASR-HIIT improves 500-m and 1,000-m paddling performances in highly trained sprint kayak athletes. Importantly, inter-subject variability (CV) of physiological adaptations to ASR-HIIT was lower than MAS-HIIT. Individualized prescription of HIIT using ASR ensures similar physiological demands across individuals and potentially facilitates similar degrees of physiological adaptation.

## 1 Introduction

High-intensity interval training (HIIT) is one of the most effective methods to improve physical performance and related physiological variables (Buchheit and Laursen, 2013; Fereshtian et al., 2017; Laursen and Buchheit, 2019; Sheykhlouvand et al., 2022). The rate at which the adaptations to HIIT occur depends on several primary variables that could be manipulated within a given HIIT session (Buchheit and Laursen, 2013). The intensity and duration of work and rest intervals are the key influencing factors (Laursen and Buchheit, 2019). Coaches attempt to optimize the training stimulus by manipulating these factors according to the specific event in which the athlete competes (Sheykhlouvand et al., 2016a). With the aim directed at ensuring athletes reach the required exercise intensity during HIIT sessions, several approaches have been developed in somewhat controlled and individualized ways (Laursen and Buchheit, 2019). Typically, HIIT is conducted at a percentage of maximal sprint speed (MSS) or maximal aerobic speed (MAS) (Julio et al., 2020). In MAS, velocity associated with the body’s maximal oxygen uptake is used as reference intensity (Sandford et al., 2021). Technically speaking, MAS is method and protocol-dependent (Laursen and Buchheit, 2019; Sandford et al., 2021). Additionally, irrespective of the method used to determine MAS, lower speed values tend to be elicited when MAS is determined using protocols with longer stage durations (Midgley et al., 2007), while larger speed increments (shorter tests) may result in higher speed values, the anaerobic capacity of the individual being the confounding variable in the assessment (Laursen and Buchheit, 2019). Also, endurance-trained athletes are likely able to tolerate longer stages and, therefore, less likely to present impairments in v/p $\dot{V}O_{2\max}$ with variations in protocol (Bentley and McNaughton, 2003). On the other hand, in practice, despite a similar MAS, two athletes can present with clearly different maximal sprint speed abilities indicating different anaerobic speed reserve (ASR) which is defined as the difference between MAS and MSS (Buchheit and Laursen, 2013). Exercise at a similar MAS will actually involve a different proportion of ASR and will result in a different physiological demand, and in turn, different exercise tolerance (Buchheit and Laursen, 2013). Hence, for individualizing training intensity, the measurement of ASR should be considered in addition to MAS during supramaximal HIIT (Laursen and Buchheit, 2019; Sandford et al., 2021). When exercising beyond MAS, what likely matters most is the degree of ASR used, rather than the relative intensity in relation to MAS (e.g., 120% vs. 140% MAS) (Blondel et al., 2001; Sandford et al., 2021). Actually, time to exhaustion at intensities above MAS is better related to the ASR and/or MSS, than to MAS (Blondel et al., 2001; Buchheit and Laursen, 2013). Moreover, when HIIT is prescribed based on the ASR, a lower variability is observed in acute physiological response during HIIT in athletes with different physiological profiles (Julio et al., 2020; Collison et al., 2021; Sandford et al., 2021). Recently, Collision and colleagues (2021) have compared the variability in supramaximal interval running performance prescribed by a proportion of MAS, ASR, and 30–15 intermittent fitness test. They found that when running speed is expressed as a percentage of the difference between MSS and MAS, inter-subject variability becomes lower than it would otherwise be. Hence, reducing supramaximal interval running performance variability ensures similar physiological demand across individuals, potentially facilitating similar degrees of physiological adaptation. However, studies currently showing the efficacy of prescribing training interventions as a proportion of ASR are limited (Sandford et al., 2021) and no previous study has examined the effects of a short period of ASR-based HIIT in sprint kayak athletes.

Sprint kayak is a race to the line on a flat water course with international competition set over four distances of 200, 500, 1,000, and 5,000 m. Races are contested as individuals and teams with up to four athletes using a double-bladed paddle used in a sitting position in a kayak (International Canoe Federation). Performance in sprint kayak requires high amounts of aerobic and anaerobic conditioning (Barzegar et al., 2021; Sheykhlouvand et al., 2022) as well as neuromuscular and mechanical contributions (García-Pallarés et al., 2009; Papandreou et al., 2020). Aerobic metabolism significantly contributes to 500, and 1,000-m performances (Bishop, 2000). Over an annual training cycle, sprint kayak athletes need to reach a peak physiological status to participate in different events and require a training program to achieve fitness in a short period of time. Improving both aerobic and anaerobic metabolism are time demanding (Rodas et al., 2000; Sheykhlouvand et al., 2022). In such situations, the apparent time-efficient aspect of HIIT might have significant implications for athletes to achieve competitive fitness in a short time frame (Sheykhlouvand et al., 2018b). Various types of HIIT programs have been shown to improve paddling performance (Yang et al., 2017; Sheykhlouvand et al., 2022). It has been reported that in events lasting 1–5 min, prescribing HIIT using ASR in workloads beyond MAS could have the strongest application (Sandford et al., 2021). Within this time frame, aerobic, anaerobic, neuromuscular, and mechanical characteristics are implemented with varying blends and large contributions to achieve optimal performance (Sandford et al., 2019). World-level kayakers complete 500, and 1,000-m sprint kayak events in ∼100 s and ∼220 s, respectively (International Canoe Federation) indicating that, HIIT prescription using ASR could be considered as an effective model for these athletes. Studies currently showing the efficacy of prescribing training interventions as a proportion of ASR are limited. Therefore, this study aimed to investigate a) if physiological and performance adaptations to ASR-based HIIT are more homogenized compared to MAS-based HIIT and b) the effects of an ASR-based HIIT performed over as brief a time period as 4 weeks on sprint kayak performance. We hypothesized that inter-subject variability (CV) of adaptations to HIIT prescribed using intensities relative to ASR will be lower compared to HIIT prescribed using MAS. In addition, our ASR-based HIIT model would improve sprint kayak performance and related physiological variables when replacing part of the traditional endurance paddling.

## 2 Materials and methods

### 2.1 Participants

Twenty-four national-level and medication-free sprint kayak athletes (age = 26 ± 6 years; Height = 181 ± 3 cm; Body mass = 81 ± 6 kg, body fat = 12 ± 2%, years of experience = 8 ± 5 years) signed an informed consent form explaining all of the procedures, risks, and benefits of the present investigation and volunteered to participate. According to a classification framework provided by McKay et al. (2022) classifying athletes as World-class, Elite/international, highly trained/national, trained/developed, recreationally active, and sedentary participants, as well as based on their performance time (see results section), our participants are considered as highly trained athletes. Following the medical screening, participants were randomly assigned to ASR-based HIIT (ASR-HIIT), MAS-based HIIT (MSA-HIIT), or a control group (CON). All procedures were in accordance with the ethical principles of the Declaration of Helsinki and approved by the local ethics committee.

### 2.2 Overview of the experimental protocol

**FIGURE 1:**
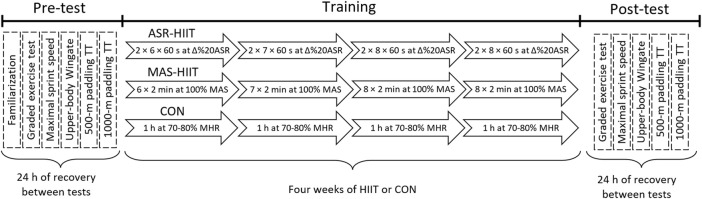
Overview of experimental protocol. HIIT, high-intensity interval training; ASR-HIIT, HIIT as a proportion of anaerobic speed reserve; MAS-HIIT, HIIT based on maximal aerobic speed, CON, traditional endurance paddling; MHR, maximum heart rate; TT, time trial. Boxes denote number, duration, and intensity of HIIT completed over a 4 week period.

Figure 1 shows an overview of the experimental protocol. Before baseline measurements, participants attended a laboratory familiarization to become oriented with all testing procedures and training protocols. In both pre- and post-training, they completed a graded exercise test to determine peak oxygen uptake $\left(\dot{V}O_{2peak}\right)$ and related physiological variables. Maximal sprint speed, repeated upper-body Wingate test, and 500 and 1,000 m paddling performances were also evaluated on separate days. Body composition was analyzed using bioimpedance (Inbody 270; Biospace Co., Ltd. Korea). All the aforementioned testing sessions were completed on different days, with 24 h of recovery separating each testing day. Two days after finishing the last training session, participants repeated the same tests under the same order and similar conditions. Participants were asked to refrain from physical activity and to record their food intake 24 h before the baseline test and then replicate their diet before and after 24 h each subsequent visit.

### 2.3 Maximal graded exercise test

A graded exercise test was performed on a kayak ergometer (SpeedStroke Gym, KayakPro, Florida, USA) that was calibrated according to the manufacturer’s instructions to determine V̇O_2peak_, MAS, V̇O_2_/HR, and ventilatory threshold [VT_1_ (%V̇O_2peak_)]. The mentioned ergometer was used during the participants’ daily program. Participants started to paddle at 6 km h^−1^, and the speed was increased by 1 km h^−1^ every minute (Sheykhlouvand et al., 2018a). The V̇O_2peak_ was established as the highest 15 s value in the test and the test was considered maximal if a plateau or slight drop in V̇O_2_ despite increased paddling speed, respiratory exchange ratio of >1.1, a HR attained ≥ 90% of age-predicted maximum HR, and physical exhaustion was met (Sheykhlouvand et al., 2016a; Sheykhlouvand et al., 2016b). During the test, expired air was continuously recorded using a breath-by-breath gas analyzer (MetaLyzer 3B-R2, Cortex, Germany) that was calibrated according to the manufacturer’s instructions. The lowest speed $\left(km\;h^{-1}\right)$ needed to elicit V̇O_2peak_ was considered as MAS. If an athlete achieved MAS during a stage that was not maintained for 1 min, the speed during the previous stage was recorded as MAS (Billat et al., 1999). VT_1_ (overall equivalent to lactate threshold) was identified as the point where an increase in both the ventilatory equivalent for oxygen (V̇_E_ V̇O_2_^−1^) and end-tidal partial pressure of oxygen (P_ET_O_2_) occurred with no concomitant increase in the ventilatory equivalent for carbon dioxide (V̇_E_ V̇CO_2_^−1^) (Alejo et al., 2022).

### 2.4 Maximal sprint speed (MSS) and ASR

Two maximal sprints with five minutes of passive recovery between them were performed on a kayak ergometer to establish the MSS (km h^−1^). After five minutes of warm-up at 65% MAS followed by three blocks of 3 s sprints and a 2 min recovery, participants were instructed to overcome the flywheel inertia from a stationary start and try to get their maximal sprint speed within ten seconds (Schram et al., 2016). Speed was tracked second by second and the highest velocity recorded by the ergometer was considered as MSS. ASR was calculated as follow:

ASR=MSS–MAS

The intensity of ASR-HIIT was calculated as follow:

Δ%20ASR=MAS+0.20×ASR

### 2.5 Upper-body wingate test

Peak power output (PPO) and average power output (APO) were assessed over four consecutive 30 s upper-body all-out tests with 4 min of passive recovery between them. Resistance was 0.075 kg/kg body mass (Forbes et al., 2014) and was preloaded onto a weight pan for immediate application at the beginning of the test. Using an arm ergometer (891E; Monark, Sweden), participants were instructed to reach maximum cranking velocity against only the ergometer’s inertial resistance over 3 s, after which the full load was applied and the electronic revolution counter activated. Verbal encouragement was provided during the 30 s trials. Using the device software, PPO and APO were calculated for each test individually. Also, we calculated the percent of the decrease in Upper-body Wingate PPO and APO from the first to last Wingate test.

### 2.6 Paddling performance

Participants completed 500−m and 1,000−m time trials on the same ergometer. Following a standardized warm-up (Borges et al., 2015), each athlete completed two trials of the 500-m test and two trials of the 1,000−m test interspersed with 1 h of passive recovery. The ergometer recorded time and the best times were used for analysis. Tests were completed on different days with 24 h between sessions.

### 2.7 Training protocols

Training started ∼48 h after the last baseline measurements. The experiment was conducted in the general preparation phase of athletes’ yearly training program. Before the experiment, all three groups had six sessions of on-water traditional endurance paddling consisting of 1 h paddling at 70%–80% HRmax. Participants who were randomly allocated to HIIT interventions (ASR-HIIT and MAS-HIIT) replaced on-water paddling sessions with supervised HIIT sessions on a kayak ergometer three times a week. In the MAS-HIIT group, participants performed six 2 min paddling intervals at 100% MAS with training volume varying each week (6, 7, 8, and 8 bouts/session from the first to the fourth week, respectively), using a 1:1 work to recovery ratio. Participants of the ASR-HIIT group completed two sets of 6 × 60 s intervals at ∆%20ASR with training volume (repetitions/set) varying each week (6, 7, 8, and 8 repetitions/set from the first to the fourth week, respectively), using a 1:2 work to recovery ratio and with 3 min of rest between sets. Each HIIT session consisted of a 10 min warm-up where participants determined the preferred speed and cadence, followed by ASR- or MAS-HIIT and 5 min cool down. The participants in the control group performed six sessions of on-water paddling per week including 60 min of endurance paddling at 70%–80% HRmax. Also, all three groups followed two sessions per week of resistance training in 3–4 sets/8–12 of repetitions/70% one repetition maximum.

### 2.8 Statistical analysis

Mean ± SD values were used for descriptive statistics. The coefficient of variation (CV) was calculated for pre-to post-training changes. Normality was tested using the Shapiro-Wilk test and Levene’s test assessed the homogeneity of variances. A 3 × 2 (group × time) repeated measure analysis of variance (ANOVA) compared the differences between groups. Tukey’s *post hoc* test analyzed the significant interactions or main effects when a significant F-ratio was observed. Pearson product–moment correlations were used to examine relationships between variables. Effect size was calculated using Cohen’s *d* (d). A commonly used interpretation is to refer to effect sizes as small $\left(d=0.2\right)$ , medium $\left(d=0.5\right)$ , and large $\left(d=0.8\right)$ based on benchmarks suggested by Cohen (1988). Statistical analyses were performed using SPSS, version 25.0 (Statistical Package for Social Science, Chicago, IL), and the level of statistical significance was set at *p* < 0.05.

## 3 Results

### 3.1 V̇O_2peak_, VT_1_, and locomotor values

**FIGURE 2:**
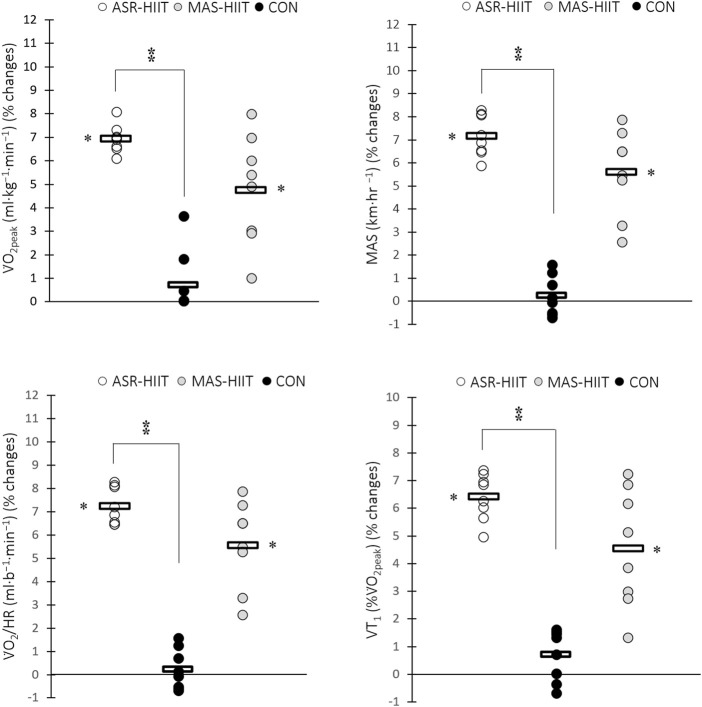
Effects of ASR-based HIIT (ASR-HIIT), HIIT based on the MAS (MAS-HIIT), and traditional endurance paddling (CON) on changes in V̇O_2peak_, maximal aerobic speed (MAS), V̇O_2_/HR, and ventilatory threshold [VT_1_ (%V̇O_2peak_)]. Circles indicate individual percent changes from baseline (X-axes) and horizontal bars represent the group mean response. N = 8 for each group. * Denotes significant difference vs. pre-training (*p* ≤ 0.05). ⁑ Denotes significant difference vs. CON group (*p* ≤ 0.05).

No pre-training difference were observed between groups for V̇O_2peak_ parameters and VT_1_. A significant time-regimen interaction was found in V̇O_2peak_ $\left(ml\;{kg}^{-1}\min^{-1}\right)$ , MAS $\left(km\;{hr}^{-1}\right)$ , V̇O_2_/HR $\left(ml\;b^{-1}∙\min^{-1}\right)$ , and VT_1_ (%V̇O_2peak_) $\left(p\;<\;0.05\right)$ . The change in V̇O_2peak_, MAS, V̇O_2_/HR, and VT_1_ in response to ASR-HIIT was significantly greater when compared to CON (*p* = 0.008, 0.001, 0.004, and 0.007, respectively). Four weeks of ASR-HIIT significantly increased V̇O_2peak_ (Pre: 50.50 ± 3.87 vs. Post: 54.04 ± 4.33 ml kg^−1^ min^−1^, %∆ = 6.9 ± 0.6, *p* = 0.00001, d = 0.8), V̇O_2_/HR (Pre: 22.6 ± 2.5 vs. Post: 24.5 ± 2.7 ml b^−1^, %∆ = 8.3 ± 1.3, *p* = 0.00001, d = 0.7), and VT_1_ (Pre: 74.6 ± 5.8 vs. Post: 79.4 ± 5.7 %V̇O_2peak_, %∆ = 6.4 ± 0.8, *p* = 0.00001, d = 0.8) (Figure 2). Also, MAS-HIIT significantly enhanced V̇O_2peak_ (Pre: 49.87 ± 4.34 vs. Post: 52.26 ± 4.71 ml kg−^1^ min^−1^, %∆ = 4.8 ± 2.3, *p* = 0.001, d = 0.5), V̇O_2_/HR (Pre: 21.1 ± 2.2 vs. Post: 22.3 ± 2.4 ml b^−1^, %∆ = 5.7 ± 2.7, *p* = 0.001, *d* = 0.5), and VT_1_ (Pre: 74.4 ± 4.8 vs. Post: 77.7 ± 5.3 %V̇O_2peak_, %∆ = 4.5 ± 2.1, *p* = 0.001, d = 0.6) pre-to post-training (Figure 2).

Pre-training individual ASR values and pre-to post-training changes in this variable are presented in Tables 1, 2, respectively. Following the training period, MAS significantly increased in ASR-HIIT group (%∆ = 7.2 ± 0.8, *p* = 0.00001, d = 1.1) and MAS-HIIT group (%∆ = 5.6 ± 1.8, *p* = 0.0004, d = .6). Also, both ASR-HIIT and MAS-HIIT interventions significantly decreased ASR (%∆ = −25.1 ± 8.1, *p* = 0.0001, d = 0.9; %∆ = −15.9 ± 8.9, *p* = 0.002, d = 1.2, respectively) pre-to post-training (Table 2).

**TABLE 1:** Pre-training anaerobic speed reserve [ASR (km·hr^−1^)] values of the participants.

Athletes	Groups		
	ASR-HIIT	MAS-HIIT	CON
1	3.33	4.45	5.86
2	4.84	4.63	5.13
3	3.53	4.01	4.98
4	6.75	5.11	4.11
5	4.87	5.62	6.32
6	5.33	5.78	4.78
7	3.45	4.01	3.65
8	4.69	5.99	5.43

**TABLE 2:** Pre-training vs. post-training values for anaerobic speed reserve (ASR), maximal sprint speed (MSS), and maximal aerobic speed (MAS).

	ASR-HIIT		MAS-HIIT		CON	
	Pre	Post	Pre	Post	Pre	Post
ASR (km·hr–1)	4.59 ± 1.15	3.50 ± 1.18	4.95 ± 0.78	4.13 ± .60	5.03 ± .87	5.05 ± 0.89
%∆	−25.1 ± 8.1		−15.9 ± 8.9		−0.5 ± 5.0	
MSS (km·hr–1)	20.47 ± 0.91	20.52 ± 0.99	20.45 ± 0.82	20.48 ± 0.92	20.53 ± 0.52	20.58 ± 0.33
%∆	+0.25 ± 1.1		+0.17 ± 1.4		+0.27 ± 1.0	
MAS (km·hr–1)	15.9 ± 0.9	17.0 ± 1.0	15.5 ± 1.3	16.3 ± 1.2	15.5 ± 1.2	15.6 ± 1.1
%∆	+7.2 ± 0.8		+5.6 ± 0.8		+0.2 ± 0.8	

Significantly greater than pre-training value (*p* < 0.05).

N = 8 for each group.

Lower coefficient of variation (CV) values was observed for the percent changes of the aforementioned factors in response to ASR-HIIT compared to MAS-HIIT for V̇O_2peak_ (8.7% vs. 48.2%), V̇O_2_/HR (15.8% vs. 48.4%), VT_1_ (13.2% vs. 47.2%), MAS (11.1% vs. 33.3%), and ASR (32.2% vs. 55.9%).

Four weeks of on-water endurance paddling had no significant effects on the aforementioned variables in CON group.

### 3.2 Upper-body wingate peak and average power output

No pre-training difference was observed between groups for Upper-body Wingate PPO and APO. Upper-body Wingate PPO in ASR-HIIT and MAS-HIIT groups significantly increased in first (*p* = 0.00002, d = .6; *p* = 0.002, d = 0.4), second (*p* = 0.0002, d = 0.7; *p* = 0.002, d = 0.3), third (*p* = 0.00004, d = 0.6; *p* = 0.0001, d = 0.4), and fourth (*p* = 0.00002, d = .6; *p* = 0.0001, d = 0.3) trials but not in CON group (Table 1). Upper-body Wingate APO increased from pre-to post-training in both ASR-HIIT and MAS-HIIT groups in first (*p* = 0.0001, d = 0.9; *p* = 0.0003, d = 0.4), second (*p* = 0.00005, d = 1.1; *p* = 0.0002, d = 0.3), third (*p* = 0.0002, d = 0.7; *p* = 0.0005, d = 0.4), and fourth (*p* = 0.00002, d = 0.7; *p* = 0.0005, d = 0.4) (Table 3).

**TABLE 3:** Pre-training vs. post-training values for upper-body peak and average power output during four consecutive wingate trials.

	ASR-HIIT		MAS-HIIT		CON	
	Pre	Post	Pre	Post	Pre	Post
PPO (W)						
1st	555.5 ± 57.4	597.7 ± 62.3	536.5 ± 72.5	566.0 ± 67.4	542.5 ± 66.1	546.6 ± 60.7
%∆	+7.6 ± 1.2		+5.7 ± 4.1		+0.8 ± 1.9	
CV (%)	16		71			
2nd	564.2 ± 52.2	604.5 ± 63.2*	541.2 ± 68.4	565.1 ± 75.6*	546.4 ± 66.7	547.6 ± 67.8
%∆	+7.0 ± 2.7		+4.3 ± 2.3		+0.2 ± 1.6	
CV (%)	38		54			
3rd	520.4 ± 73.2	565.4 ± 78.7*	514.2 ± 67.1	540.5 ± 72.2	513.0 ± 67.8	515.6 ± 60.7
%∆	+8.6 ± 1.8		+5.1 ± 1.7		+0.7 ± 3.1	
CV (%)	21		33			
4th	491.6 ± 63.5	529.6 ± 70.1*	472.0 ± 66.7	493.4 ± 67.8	479.1 ± 76.5	482.5 ± 76.0
%∆	+7.7 ± 0.8		+4.6 ± 1.7		+0.7 ± 1.8	
CV (%)	10		38			
APO (W)						
1st	386.1 ± 32.1	415.9 ± 33.4	369.7 ± 55.2	390.6 ± 58.2*	361.4 ± 41.5	362.5 ± 37.8
%∆	+7.7 ± 0.8		+5.7 ± 1.7		+0.4 ± 2.7	
CV (%)	10		30			
2nd	396.5 ± 33.0	434.6 ± 37.5*	374.9 ± 55.2	395.1 ± 61.8	379.4 ± 42.9	382.6 ± 45.4
%∆	+9.6 ± 1.2		+5.3 ± 1.7		+0.8 ± 2.1	
CV (%)	12		54			
3rd	369.6 ± 45.3	404.7 ± 49.4*	358.7 ± 46.8	379.4 ± 50.6	355.5 ± 40.2	357.4 ± 34.9
%∆	+9.5 ± 0.9		+5.4 ± 1.5		+0.7 ± 1.6	
CV (%)	9		27			
4th	344.0 ± 42.8	377.9 ± 47.6	330.2 ± 43.2	347.9 ± 46.3	333.2 ± 49.6	335.4 ± 49.7
%∆	+9.8 ± 1.3		+5.3 ± 1.5		+0.7 ± 1.7	
CV (%)	13		27			

HIIT, high-intensity interval training; ASR-HIIT, HIIT, based on anaerobic speed reserve; MAS-HIIT, HIIT, based on maximal aerobic speed; PPO, peak power output; APO, average power output; CV, coefficient of variation; * Significantly greater than pre-training value (*p* < 0.05).

N = 8 for each group.

Four weeks of ASR-HIIT didn’t change the percent of the decrease in Upper-body Wingate PPO from the first to last Wingate test (Pre: 13.6 ± 9.0% vs. Post: 13.4 ± 8.9%). However, the percent of the decrease in Upper-body Wingate APO from the first to last Wingate test significantly attenuated in response to ASR-HIIT (Pre: 12.9% ± 8.8 vs. Post: 10.7 ± 8.5%, *p* = 0.007, d = .3). No significant changes took place in MAS-HIIT and CON groups.

The coefficient of variation of percent changes in PPO for the ASR-HIIT group in the first (16%), second (38%), third (21%), and fourth (10%) trials was lower than that of the MAS-HIIT group (71%, 54%, 33%, and 38% from first to fourth trials, respectively). Also, CV values of percent changes in PPO in response to ASR-HIIT in the first (10%), second (12%), third (9%), and fourth (13%) trials was lower than MAS-HIIT group (30%, 54%, 27%, and 27% from first to fourth trials, respectively).

### 3.3 500-m and 1000-m time trial

**FIGURE 3:**
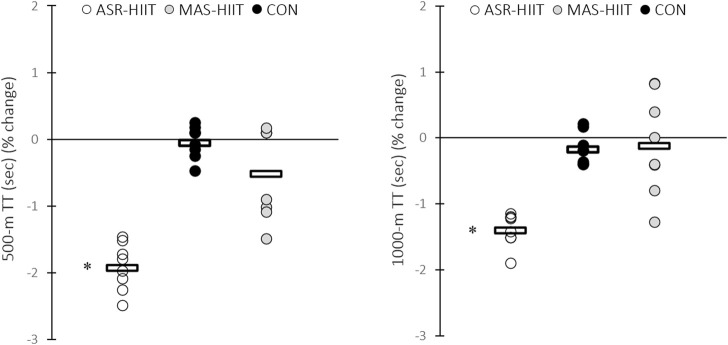
Effects of ASR-based HIIT (ASR-HIIT), HIIT based on the MAS (MAS-HIIT), and traditional endurance paddling (CON) on 500-m, and 1000-m time trial performance. Circles indicate individual percent changes from baseline (X-axes) and horizontal bars represent the group mean response. N = 8 for each group. * Denotes significant difference vs. pre-training (*p* ≤ 0.05).

No pre-training difference was observed between groups for 500-m and 1000-m time trial performance. Figure 3 shows performance changes in different training groups. 500-m paddling time significantly decreased in response to ASR-HIIT (Pre: 120.37 ± 4.37 vs. Post: 118.05 ± 3.88 s, %∆ = −1.92, *p* = 0.00004, d = 0.56) but not in MAS-HIIT (Pre: 124.25 ± 4.26 vs. Post: 123.61 ± 4.47 s, %∆ = −0.29, *p* = 0.17) and CON (Pre: 121.87 ± 3.22 vs. Post: 121.81 ± 3.34 s, %∆ = −0.16, *p* = 0.57) groups. Also, ASR-HIIT resulted in significant decrease in 1000 m paddling time (Pre: 244.62 ± 6.78 vs. Post: 241.08 ± 6.95 s, %∆ = −1.5, *p* = 0.00001, d = 0.51) pre-to post-training and this variable remained unchanged in response to MAS-HIIT (Pre: 243.75 ± 6.11 vs. Post: 243.05 ± 7.00 s, %∆ = −0.11, *p* = 0.16) and CON (Pre: 246.00 ± 3.96 vs. Post: 245.58 ± 4.35 s, %∆ = −0.17, *p* = 0.11). No between-group difference was found in 500-m and 1000-m time trial performances. CV values for the percent changes of 500-m and 1000-m in response to ASR-HIIT were 18.7% and 26.4%, respectively.

Also, 500- and 1,000-m paddling performances were negatively correlated to V̇O_2peak_ (*r* = −0.71, *p* = 0.0001; *r* = −0.69, *p* = 0.0001, respectively), MAS (*r* = −0.66, *p* = .0004; *r* = −0.54, *p* = 0.006, respectively), and MSS (*r* = −0.42, *p* = 0.04; *r* = −.81, *p* = 0.00002, respectively).

## 4 Discussion

The primary findings in the present study support our stated hypothesis that inter-subject variability (CV) of adaptations to HIIT prescribed using intensities relative to ASR is decreased compared to the prescription using the MAS in athletes with different ASRs. Also, ASR-HIIT improves 500-m and 1,000-m paddling performance and related physiological variables compared to MAS-HIIT.

To the best of our knowledge, this study is the first to examine the homogeneity of adaptations in physiological and performance parameters in response to a paddling-based HIIT prescribed using anaerobic speed reserve. These results corroborate the propositions provided by the previous studies (Julio et al., 2020; Collison et al., 2021), suggesting anaerobic speed reserve is an invaluable factor in the individualized prescription of HIIE intensity. Previous studies indicated that the decrease in the CV in performance represents a more matched physiological cost for individuals allowing them to design effective training programs (Julio et al., 2020).

In the first study on ASR, Blondel and colleagues (2001) examined the inter-individual variability in running time to exhaustion when running speed was expressed as a percentage of the difference between a) MSS and critical velocity; b) MAS and critical velocity; and c) MSS and MAS. Their results showed that the same intensity relative to aerobic contribution doesn’t represent the same absolute intensity for all and could partly explain variability in time to exhaustion. Hence, expressing intensity as a percentage of ASR for supra-maximal velocities allows individual differences in anaerobic work capacity to be taken into account. Recently, Collison and colleagues (2021) performed supramaximal interval running trials (15 s on: 15 s off until volitional exhaustion) at 120% MAS, 20% ASR, and 95% 30–15 intermittent fitness test in a randomized order and they calculated variability in time to exhaustion for each prescription method. Their results showed that time to exhaustion residuals is reduced when prescribed by ASR compared with MAS (standardized mean difference [SMD] = −0.47; 29%). Although confidence intervals about this reduction indicated that there was some uncertainty in this finding (SMD = −1.03 to 0.09; *p* = 0.09), they mentioned that the 29% reduction exceeds the inherent error in time to exhaustion efforts (i.e., ∼9%–15%) and may thus be considered practically meaningful.

Consistent with these studies, Figures 2, 3 indicate wide dispersion of pre-to post-training changes in response to MAS-HIIT when compared with that of ASR-HIIT. Lower CV values of percent changes in all physiological parameters and performance in response to ASR-HIIT could in part be a result of uniform physiological stress and subsequent adaptation across diverse athlete profiles. It could be a key rationale that ASR-HIIT could be considered as a new training stimulus to impose uniform physiological adaptations. However, the only difference between our HIIT protocols was not in how the intensity was prescribed and the greater difficulty of ASR-HIIT sessions compared to MAS-HIIT should also be considered. Hence, it is ambiguous to conclude whether a lower CV comes from how the intensity was prescribed.

In terms of exercise performance, it has previously been observed that increased middle-distance sprint kayak performance is accompanied by an increase in aerobic power (O_2_ delivery and extraction/consumption by the muscle) (Paquette et al., 2021), anaerobic metabolism (Papandreou et al., 2020), anaerobic power (Sheykhlouvand et al., 2022). Although these parameters significantly increased in both HIIT groups, 500-m and 1,000-m paddling performances only improved in the ASR-HIIT group. Both training programs differed in work interval duration and intensity and work-to-recovery ratio. Since the time spent at target intensity was the same between programs, it is likely that the ASR-HIIT sessions were of higher difficulty. Hence, this is also possible that the greater adaptations following the ASR-HIIT program be in part a result of the higher level of difficulty, or higher load, associated with these sessions. Buffering capacity also plays a key role in improving exercise performance. Metabolite accumulation has long been considered one of the factors contributing to reduced exercise performance and capacity with the accumulation of hydrogen ions (H^+^), which causes acidification in the muscle, associated with muscle fatigue (Lancha Junior et al., 2015). However, we haven’t analyzed this factor.

V̇O_2peak_ and ventilatory threshold are among the main physiological parameters affecting 500 and 1,000-m sprint kayak performance (Sheykhlouvand et al., 2022; Papandreou et al., 2020; Paquette et al., 2018 and Paquette et al., 2021; Borges et al., 2015; García-Pallarés et al., 2010). The increase in V̇O_2peak_ observed in this study (ASR-HIIT: 6.9 ± 0.6%; MAS-HIIT: 4.8 ± 2.3%) was in agreement with that of previous studies that employed different short-term HIIT regimens (Rønnestad et al., 2020; Sheykhlouvand et al., 2018a,b). Our finding support Sheykhlouvand et al. (2018a) who reported increased V̇O_2peak_ (L·min^−1^) (8.8%) in response to a 3-week paddling-based HIIT in professional paddlers (6 × 1 min at 100% *v*V̇O_2max_ wit variable volume 1:3 work to recovery ratio). In another study, they indicated that 4 weeks of sprint interval training (3–6 sets of 5 × 5 s “all out” paddling, interspersed with 3 min of rest between efforts) improves V̇O_2peak_ (ml kg^−1^ min^−1^) (7.6%) in well-trained paddlers (Sheykhlouvand et al., 2018b). Likewise, Rønnestad and colleagues (2019) showed that V̇O_2max_ (mlkg^−1^ min^−1^) was increased (3%) after 3 weeks of HIIT (3 series with 13 × 30 s work intervals interspersed with 15 s recovery and three in recovery between series) in elite cyclists. Increases in oxygen delivery (i.e., central component) and oxygen use by active muscles (i.e., peripheral component) are the main proposed mechanisms involved in V̇O_2peak_ changes (Laursen and Buchheit, 2019; Dolci et al., 2020; Sayevand et al., 2022). Improved central component (cardiac function) in our participants is supported by enhanced V̇O_2_/HR which is considered a good means of appraising indirectly stroke volume in trained participants (Sheykhlouvand et al., 2018b; Bernardi et al., 2020). Although we haven’t evaluated peripheral mechanisms, it has been noted that capillarization and mitochondrial content have a remarkable effect on V̇O_2peak_ (Sheykhlouvand et al., 2018b; Dolci et al., 2020). Exercise-induced increases in capillary density might require months to occur. Non-etheless, evidence suggests an increase in mitochondrial content can occur quickly after HIIT (Rodas et al., 2000) leading to improvements in maximal aerobic capacity (Dolci et al., 2020).

VT_1_ (overall equivalent to lactate threshold) is established as an essential indicator of exercise intensity tolerance and a good marker of one’s ability to perform higher intensities of exercise for longer durations (Farah et al., 2015). Improved VT_1_ in our participants was in line with previous studies reporting an improvement in VT_1_ following paddling-based HIIT (Papandreou et al., 2020; Sheykhlouvand et al., 2016b).

Other important adaptations after the training period were an increased MAS and decreased ASR in response to both HIIT protocols. Our data indicated both 500- and 1,000-m performances are negatively correlated to V̇O_2peak_, MAS, and MSS. Since MSS remained unchanged pre-to post-training, a decrease in the ASR can be attributed to an increase in MAS. Our HIIT protocols were performed at 100% MAS (MAS-HIIT) and at the lower bound of ASR (20%) (ASR-HIIT), both of which could have a remarkable effect on MAS. Improved movement economy (Billat et al., 1999), neural adaptations (Creer et al., 2004), ventilatory threshold (Sheykhlouvand et al., 2016b), and exercise tolerance (Demarle et al., 2003) are possible explanations for enhanced MAS.

Enhanced PPO and APO in repeated upper-body Wingate tests was another important adaptation to HIIT interventions. Upper-body PPO and APO are strong determinants of paddling performance (Sheykhlouvand and Forbes, 2017). Augmenting the pulling motion through increasing stroke power enhances the pulling force of paddling and enhanced maintenance of power (APO) during the race improves the maintenance of speed. Enhanced peak and average power output is commonly observed in the majority of studies employing short-term HIIT in athletes. Farzad et al. (2011) indicated that 4 weeks of running HIIT (6 × 35 m all-out running with 10 s recovery between efforts) increases both PPO and APO. Laursen, (2010) have reported that power output increases following only 2 weeks of HIIT in well-trained cyclists (20 × 1-m at V̇O_2peak_ PO with 2 min recovery at 50 W). Also, in a paddling-based HIIT, Sheykhlouvand and colleagues (2018b, and 2016a) indicated that PPO and APO are improved after a short period of time (3–4 weeks). Increased discharge rate and recruitment of high-threshold motor units (Dolci et al., 2020), enhanced total creatin content of active muscles (Hoffmann et al., 2020), and improved muscle buffering capacity (Sheykhlouvand et al., 2018b) are possible explanations for our findings.

A limitation of this study was the inability to strictly monitor the dietary practices of athletes during training. Moreover, we only recruited men, and our results cannot be applied to women competing in sprint kayaking. Our results only apply to our specific HIIT regimens, and it is unknown if similar adaptations would occur in response to higher or lower volumes of HIIT.

In conclusion, data suggest that 4 weeks of HIIT prescription as a proportion of anaerobic speed reserve improves middle-distance sprint kayak performance and related physiological variables. Also, the adaptations to ASR-based HIIT are more homogenous when compared to HIIT prescribed using MAS under the conditions of this study. This is the first study to indicate a practical model of ASR-HIIT for sprint kayak athletes. Our results indicated replacing part of traditional endurance paddling sessions with this HIIT model could be considered as a useful method for athletes to achieve competitive fitness in a short time frame. More importantly, our findings suggest that using the athlete’s ASR may help to better guide sprint kayak athletes and their coaches in choosing a more individualized training load.

## Data Availability

The raw data supporting the conclusions of this article will be made available by the authors, without undue reservation.

## References

[B1] Alejo L. B. Montalvo-Pérez A. Valenzuela P. L. Revuelta C. Ozcoidi L. M. de la Calle V. (2022). Comparative analysis of endurance, strength and body composition indicators in professional, under-23 and junior cyclists. Front. Physiol. 13, 945552. 10.3389/fphys.2022.945552 35991188 PMC9388719

[B2] Barzegar H. Arazi H. Mohsebbi H. Sheykhlouvand M. Forbes S. C. (2021). Caffeine co-ingested with carbohydrate on performance recovery in national level paddlers: A randomized, double-blind, crossover, placebo-controlled trial. J. Sport. Med. Phys. Fit. 62, 337–342. 10.23736/S0022-4707.21.12125-5 34498818

[B3] Bentley D. J. McNaughton L. R. (2003). Comparison of W(peak), VO_2_(peak) and the ventilation threshold from two different incremental exercise tests: Relationship to endurance performance. J. Sci. Med. Sport. 6 (4), 422–435. 10.1016/s1440-2440(03)80268-2 14723392

[B4] Bernardi M. Guerra E. Rodio A. Dante D. Castellani V. Peluso I. (2020). Assessment of exercise stroke volume and its prediction from oxygen pulse in paralympic athletes with locomotor impairments: Cardiac long-term adaptations are possible. Front. Physiol. 10, 1451. 10.3389/fphys.2019.01451 32218739 PMC7079670

[B5] Billat V. L. Flechet B. Petit B. Muriaux G. Koralsztein J. P. (1999). Interval training at VO_2max_: Effects on aerobic performance and overtraining markers. Med. Sci. Sports. Exerc. 31, 156–163. 10.1097/00005768-199901000-00024 9927024

[B46] Bishop D. (2000). Physiological predictors of flat-water kayak performance in women. Eur. J. Appl. Physiol. 82 (1-2), 91–97. 10.1007/s004210050656 10879448

[B6] Blondel N. Berthoin S. Billat V. Lensel G. (2001). Relationship between run times to exhaustion at 90, 100, 120, and 140% of vVO_2_max and velocity expressed relatively to critical velocity and maximal velocity. Int. J. Sports. Med. 22 (1), 27–33. 10.1055/s-2001-11357 11258638

[B7] Borges T. O. Dascombe B. Bullock N. Coutts A. J. (2015). Physiological characteristics of well-trained junior sprint kayak athletes. Int. J. Sports. Physiol. Perform. 10, 593–599. 10.1123/ijspp.2014-0292 25473923

[B8] Buchheit M. Laursen P. B. (2013). High-intensity interval training, solutions to the programming puzzle: Part I: Cardiopulmonary emphasis. Sports. Med. 43, 313–338. 10.1007/s40279-013-0029-x 23539308

[B9] Cohen J. (1988). Statistical power analysis for the behavioral sciences. New York, NY: Routledge Academic.

[B10] Collison J. Debenedictis T. Fuller J. T. Gerschwitz R. Ling T. Gotch L. (2021). Supramaximal interval running prescription in Australian rules football players: A comparison between maximal aerobic speed, anaerobic speed reserve, and the 30-15 intermittent fitness test. J. Strength. Cond. Res. 36 (12), 3409–3414. 10.1519/JSC.0000000000004103 34387223

[B11] Creer A. R. Ricard M. D. Conlee R. K. Hoyt G. L. Parcell A. C. (2004). Neural, metabolic, and performance adaptations to four weeks of high intensity sprint-interval training in trained cyclists. Int. J. Sports. Med. 25, 92–98. 10.1055/s-2004-819945 14986190

[B12] Demarle A. P. Heugas A. M. Slawinski J. J. Tricot V. M. Koralsztein J. P. Billat V. L. (2003). Whichever the initial training status, any increase in velocity at lactate threshold appears as a major factor in improved time to exhaustion at the same severe velocity after training. Arch. Physiol. Biochem. 111, 167–176. 10.1076/apab.111.2.167.14003 12919004

[B13] Dolci F. Kilding A. E. Chivers P. Piggott B. Hart N. H. (2020). HighIntensity interval training shock microcycle for enhancing sport performance: A brief review. J. Strength. Cond. Res. 34, 1188–1196. 10.1519/JSC.0000000000003499 31904712

[B14] Farah B. Q. Ritti-Dias R. M. Cucato G. G. Meneses A. L. Gardner A. W. (2015). Clinical predictors of ventilatory threshold achievement in claudicants. Med. Sci. Sports Exerc 47, 493–497. 10.1249/MSS.0000000000000434 25003779 PMC4286519

[B15] Farzad B. Gharakhanlou R. Agha-Alinejad H. Curby D. G. Bayati M. Bahraminejad M. (2011). Physiological and performance changes from the addition of a sprint interval program to wrestling training. J. Strength. Cond. Res. 25, 2392–2399. 10.1519/JSC.0b013e3181fb4a33 21849912

[B16] Fereshtian S. Sheykhlouvand M. Forbes S. Agha-Alinejad H. Gharaat M. (2017). Physiological and performance responses to high-intensity interval training in female inline speed skaters. Apunts. Med. L’esport. 52, 131–138. 10.1016/j.apunts.2017.06.003

[B17] Forbes S. C. Kennedy M. D. Boule N. B. Bell G. (2014). Determination of the optimal load setting for arm crank anaerobic testing in men and women. Int. J. Sports. Med. 35, 835–839. 10.1055/s-0034-1368789 24920563

[B18] García-Pallarés J. Garcia-Fernandez M. Sanchez-Medina L. Izquierdo, M. (2010). Performance changes in world-class kayakers following two different training periodization models. Eur. J. Appl. Physiol. 110 (1), 99–107. 10.1007/s00421-010-1484-9 20414669

[B19] García-Pallarés J. Sanchez-Medina L. Carrasco L. Diaz A. Sánchez-Medina L. Izquierdo M. (2009). Endurance and neuromuscular changes in world-class level kayakers during a periodized training cycle. Eur. J. Appl. Physiol. 106 (4), 629–638. 10.1007/s00421-009-1061-2 19396614

[B20] Hoffmann S. M. Skinner T. L. van Rosendal S. P. Osborne M. A. Emmerton L. M. Jenkins D. G. (2020). The efficacy of the lactate threshold: A sex-based comparison. J. Strength. Cond. Res. 34, 3190–3198. 10.1519/JSC.0000000000002654 33105370

[B21] Julio U. F. Valéria L G Panissa V. L. G. Paludo A. C. Alves E. D. Campos F. A. D. Franchini E. (2020). Use of the anaerobic speed reserve to normalize the prescription of high-intensity interval exercise intensity. Eur. J. Sport. Sci. 20 (2), 166–173. 10.1080/17461391.2019.1624833 31132025

[B22] Lancha Junior A. H. de Salles Painelli V. Saunders B. Artioli G. G. (2015). Nutritional strategies to modulate intracellular and extracellular buffering capacity during high-intensity exercise. Sports. Med. 45, 71–81. 10.1007/s40279-015-0397-5 26553493 PMC4672007

[B23] Laursen P. B. Buchheit M. (2019). in Science and application of high-intensity interval training. 1st Edn (Champaign: Human Kinetics), 119.

[B24] Laursen P. B. (2010). Training for intense exercise performance: Highintensity or high-volume training? Scand. J. Med. Sci. Sports. 2, 1–10. 10.1111/j.1600-0838.2010.01184.x 20840557

[B26] McKay A. K. A. Stellingwerff T. Smith E. S. Martin D. T. Mujika I. Goosey-Tolfrey V. L. (2022). Defining training and performance caliber: A participant classification framework. Int. J. Sports. Physiol. Perform. 17 (2), 317–331. 10.1123/ijspp.2021-0451 34965513

[B27] Midgley A. W. McNaughton L. R. Carroll S. (2007). Time at V̇O_2max_ during intermittent treadmill running: Test protocol dependent or methodological artefact? Int. J. Sports. Med. 28 (11), 934–939. 10.1055/s-2007-964972 17497578

[B28] Papandreou A. Philippou A. ZacharogiannisPhilippou E. A. Zacharogiannis E. Maridaki. M. (2020). Physiological adaptations to high-intensity interval and continuous training in kayak athletes. Cond. Res. 34 (8), 2258–2266. 10.1519/JSC.0000000000002710 29952869

[B29] Paquette M. Bieuzen F. Billaut F. (2018). Muscle oxygenation rather than V̇O_2max_ as a strong predictor of performance in sprint canoe-kayak. Int. J. Sports. Physiol. Perform. 19, 1299–1307. 10.1123/ijspp.2018-0077 29745773

[B30] Paquette M. Bieuzen F. Billaut F. (2021). The effect of HIIT vs. SIT on muscle oxygenation in trained sprint kayakers. Eur. J. Appl. Physiol. 121 (10), 2743–2759. 10.1007/s00421-021-04743-z 34145486

[B31] Rodas G. Ventura J. L. Cadefau J. A. Cusso´ R. Parra J. (2000). A short training programme for the rapid improvement of both aerobic and anaerobic metabolism. Eur. J. Appl. Physiol. 82, 480–486. 10.1007/s004210000223 10985604

[B32] Rønnestad B. R. Hansen J. Nygaard H. Lundby C. (2020). Superior performance improvements in elite cyclists following short-interval vs effort-matched long-interval training. Scand. J. Med. Sci. Sports 30 (5), 849–857. 10.1111/sms.13627 31977120

[B33] Sandford G. N. Allen S. V. Kilding A. E. Ross A. Laursen P. B. (2019). Anaerobic speed reserve: A key component of elite male 800-m running. Int. J. Sports. Physiol. Perform. 14, 501–508. 10.1123/ijspp.2018-0163 30300023

[B34] Sandford G. N. Laursen P. B. Buchheit M. (2021). Anaerobic speed/power reserve and sport performance: Scientific basis, current applications and future directions. Sports. Med. 51 (10), 2017–2028. 10.1007/s40279-021-01523-9 34398445

[B36] Sayevand Z. Nazem F. Nazari A. Sheykhlouvand M. Forbes S. C. (2022). Cardioprotective effects of exercise and curcumin supplementation against myocardial ischemia–reperfusion injury. Sport. Sci. Health. 18, 1011–1019. 10.1007/s11332-021-00886-w

[B37] Schram B. Hing W. Climstein M. (2016). Profiling the sport of stand-up paddle boarding. J. Sports. Sci. 34 (10), 937–944. 10.1080/02640414.2015.1079331 26289320

[B38] Sheykhlouvand M. Arazi H. Astorino T. A. Suzuki K. (2022). Effects of a new form of resistance-type high-intensity interval training on cardiac structure, hemodynamics, and physiological and performance adaptations in well-trained kayak sprint athletes. Front. Physiol. 13, 850768. 10.3389/fphys.2022.850768 35360225 PMC8960736

[B39] Sheykhlouvand M. Forbes S. C. (2017). Aerobic capacities, anaerobic power, and anthropometric characteristics of elite female canoe polo players based on playing position. Sport. Sci. Health. 14, 19–24. 10.1007/s11332-017-0395-0

[B40] Sheykhlouvand M. Gharaat M. Khalili E. Agha-Alinejad H. (2016b). The effect of high-intensity interval training on ventilatory threshold and aerobic power in well-trained canoe polo athletes. Sci. Sports. 31, 283–289. 10.1016/j.scispo.2016.02.007

[B41] Sheykhlouvand M. Gharaat, 5 Khalili M. Agha-Alinejad E. Rahmaninia H. Arazi F. (2018a). Low-volume high-intensity interval versus continuous endurance training: Effects on hematological and cardiorespiratory system adaptations in professional canoe polo athletes. J. Strength. Cond. Res. 32, 1852–1860. 10.1519/JSC.0000000000002112 28700514

[B42] Sheykhlouvand M. Khalili E. Agha-Alinejad H. Gharaat M. A. (2016a). Hormonal and physiological adaptations to high-intensity interval training in professional male canoe polo athletes. J. Strength. Cond. Res. 30, 859–866. 10.1519/JSC.0000000000001161 26349044

[B43] Sheykhlouvand M. Khalili E. Gharaat M. Arazi H. Khalafi M. Tarverdizadeh B. (2018b). Practical model of low-volume paddling-based sprint interval training improves aerobic and anaerobic performances in professional female canoe polo athletes. J. Strength. Cond. Res. 32, 2375–2382. 10.1519/JSC.0000000000002152 29239986

[B44] Yang M. T. Lee M M. Hsu S. C. Chan K. H. (2017). Effects of high-intensity interval training on canoeing performance. Eur. J. Sport. Sci. 17 (7), 814–820. 10.1080/17461391.2017.1314553 28445078

